# Molecular genomic and epigenomic characteristics related to aspirin and clopidogrel resistance

**DOI:** 10.1186/s12920-024-01936-1

**Published:** 2024-06-20

**Authors:** Jei Kim, Byoung-Soo Shin, Dae-Hyun Kim, Dong-Ick Shin, Seong Hwan Ahn, Jae Guk Kim, Su Hyun Ryu, Hye Rin Moon, Hyun Goo Kang, Hyeseon Jeong, Kyu Sun Yum, Hee-Yun Chae, Do-Hyung Kim, Keunsoo Kang, Jeeyeon Kim

**Affiliations:** 1 Department of Neurology, College of Medicine and Hospital, Daejeon-Chungnam Regional Cardiocerebrovascular Disease Center, Chungnam National University, Daejeon, South Korea; 2 Department of Neurology, Research Institute of Clinical Medicine and Biomedical Research Institute, Medical School and Hospital, Jeonbuk National University, Jeonju, South Korea; 3 Department of Neurology, Busan Regional Cardiocerebrovascular Disease Center, Dong-A University Hospital, Busan, South Korea; 4 Department of Neurology, Chungbuk Regional Cardiocerebrovascular Disease Center, Chungbuk National University Hospital, Cheongju, South Korea; 5 Department of Neurology, Chosun University Hospital, Gwangju, South Korea; 6 Department of Neurology, Eulji University Hospital, Daejeon, South Korea; 7 Department of Microbiology, College of Science & Technology, Dankook University, Cheonan, South Korea; 8 Department of Anatomy, College of Medicine, Chungnam National University, 266 Moonhwaro, Joongku, Daejeon, 35015 South Korea

**Keywords:** Aspirin resistance, Clopidogrel resistance, Genomic characteristics, Epigenomic characteristics, Arachidonic acid metabolism, Clopidogrel activation

## Abstract

**Background:**

Mediators, genomic and epigenomic characteristics involving in metabolism of arachidonic acid by cyclooxygenase (COX) and lipoxygenase (ALOX) and hepatic activation of clopidogrel have been individually suggested as factors associated with resistance against aspirin and clopidogrel. The present multi-center prospective cohort study evaluated whether the mediators, genomic and epigenomic characteristics participating in arachidonic acid metabolism and clopidogrel activation could be factors that improve the prediction of the aspirin and clopidogrel resistance in addition to cardiovascular risks.

**Methods:**

We enrolled 988 patients with transient ischemic attack and ischemic stroke who were evaluated for a recurrence of ischemic stroke to confirm clinical resistance, and measured aspirin (ARU) and P2Y12 reaction units (PRU) using VerifyNow to assess laboratory resistance 12 weeks after aspirin and clopidogrel administration. We investigated whether mediators, genotypes, and promoter methylation of genes involved in COX and ALOX metabolisms and clopidogrel activation could synergistically improve the prediction of ischemic stroke recurrence and the ARU and PRU levels by integrating to the established cardiovascular risk factors.

**Results:**

The logistic model to predict the recurrence used thromboxane A synthase 1 (*TXAS1*, rs41708) A/A genotype and *ALOX12* promoter methylation as independent variables, and, improved sensitivity of recurrence prediction from 3.4% before to 13.8% after adding the mediators, genomic and epigenomic variables to the cardiovascular risks. The linear model we used to predict the ARU level included leukotriene B4, *COX2* (rs20417) C/G and thromboxane A2 receptor (rs1131882) A/A genotypes with the addition of *COX1* and *ALOX15* promoter methylations as variables. The linear PRU prediction model included G/A and prostaglandin I receptor (rs4987262) G/A genotypes, *COX2* and *TXAS1* promoter methylation, as well as cytochrome P450 2C19*2 (rs4244285) A/A, G/A, and *3 (rs4986893) A/A genotypes as variables. The linear models for predicting ARU (*r* = 0.291, R^2^ = 0.033, *p* < 0.01) and PRU (*r* = 0.503, R^2^ = 0.210, *p* < 0.001) levels had improved prediction performance after adding the genomic and epigenomic variables to the cardiovascular risks.

**Conclusions:**

This study demonstrates that different mediators, genomic and epigenomic characteristics of arachidonic acid metabolism and clopidogrel activation synergistically improved the prediction of the aspirin and clopidogrel resistance together with the cardiovascular risk factors.

**Trial registration:**

URL: https://www.clinicaltrials.gov; Unique identifier: NCT03823274.

**Supplementary Information:**

The online version contains supplementary material available at 10.1186/s12920-024-01936-1.

## Background

Aspirin and clopidogrel are the most widely used medical treatments for reducing non-cardioembolic stroke recurrence [1]. However, 15–50% of patients with recurrent ischemic stroke are already on antiplatelet medications [1]. Therefore, failure to prevent ischemic stroke recurrence following aspirin and clopidogrel administration is a major clinical challenge [1]. 

The prevalence of antiplatelet resistance ranges from 3- 65%, 8–56%, and 1.8–35% for aspirin, clopidogrel, and aspirin combined with clopidogrel, respectively, in patients with ischemic stroke [2], even though the resistances to aspirin and clopidogrel differently measured using the clinical resistance checked by recurrence of ischemic stroke or the laboratory resistance measured by platelet reactivity changes after administration of the antiplatelets in previous studies [3]. More studies are required to identify factors related to the clinical and laboratory resistance by integrating molecular genomic and epigenomic characteristics of the metabolic and activation pathways of aspirin and clopidogrel with the established cardiovascular risk factors [4, 5]. 

Pharmacodynamic shunting between cyclooxygenase (COX) and arachidonate lipoxygenase (ALOX) metabolism of arachidonic acid and pharmacokinetic activation of the prodrug clopidogrel has been targeted to identify factors related to aspirin and clopidogrel resistance [6, 7]. Single nucleotide polymorphisms (SNPs) of the genes participating in the COX [8] and ALOX [9] metabolic pathways and involved in the hepatic metabolism of clopidogrel [7] have been studied to elucidate the pharmacodynamic and pharmacokinetic characteristics related to aspirin and clopidogrel resistance. Environmental risk factors such as aging, smoking, and hypercholesterolemia also cause resistance to these drugs [1]. Accumulation of the risk factor effects throughout aging results in gene silencing without genetic polymorphisms [10, 11] This occurs through epigenetic alterations such as gene-specific promoter DNA methylation changes [10, 11]. Studies have recently attempted to identify epigenomic characteristics related to aspirin [12] and clopidogrel resistance [13]. However, the individual gene-specific genomic and epigenomic characteristics outlined in the previous studies have not been well documented as potential markers of aspirin and clopidogrel resistance by integrating with the cardiovascular risk factors.

In the present study, we aimed to investigate whether mediators, SNPs, and promoter methylation of the genes participating in COX and ALOX metabolism and cytochrome P450 2C19 (*CYP2C19*) activation could be factors to predict the aspirin and clopidogrel resistance together with the cardiovascular risk factors in patients with ischemic stroke.

## Methods

### STRAPER study

To evaluate mediators, genomic and epigenomic characteristics related to aspirin and clopidogrel resistance, we conducted a prospective cohort clinical trial titled ‘Multi-center, prospective, cohort study to evaluate the relationship of STroke Recurrence and Anti-PlatElet Resistance in ischemic stroke patients’ (STRAPER study; ClinicalTrials.gov Identifier, NCT03823274; funding support, Yuhan Corporation, South Korea). Six hospitals participated in the study: Chungnam National University Hospital, Daejeon; Jeonbuk National University Hospital, Jeonju; Dong-A University Hospital, Busan; Chungbuk National University Hospital, Cheongju; Chosun University Hospital, Gwangju; and Eulji University Hospital, Daejeon, South Korea.

The STRAPER study prospectively screened 1,011 patients who were ≥ 50 years old and diagnosed with transient ischemic attack (TIA) and acute ischemic strokes from December 31, 2018 to December 31, 2021 across six hospitals, and enrolled 1,002 patients after review of the inclusion criteria for the study. TIA was defined as a transient neurological deficit lasting < 24 h with no acute lesion observed within 72 h of symptom onset, as well as acute ischemic strokes with new lesions detected on diffusion-weighted magnetic resonance imaging. Patients who were diagnosed with atrial fibrillation before and after admission were excluded from the study. We collected the cardiovascular risk factors and fasting blood test results obtained within 24 h after admission for the individual patients (Table 1).

**Table 1:** Comparisons of the variables of patients with or without recurrence of ischemic stroke

Variable groups	Variables	Ischemic stroke recurrence		Total (*n* = 988)	*p*-value
		No (*n* = 959)	Yes (*n* = 29)		
Risk factors	Age (years, ± SD)	69.0 ± 9.3	65.9 ± 9.1	68.9 ± 9.3	0.071
	Sex (men: women, %)	620 (65):339 (35)	24 (83):5 (17)	644 (65):344 (35)	0.044
	Hypertension (%)	570 (59.4)	19 (65.5)	589 (59.6)	0.511
	Diabetes (%)	299 (31.2)	13 (44.8)	312 (31.6)	0.119
	Smoking (%)	242 (25.2)	17 (58.6)	259 (26.2)	< 0.001
Platelet function tests (average)	ARU	476.1 ± 57.2	464.1 ± 53.7	475.7 ± 57.1	0.267
	PRU	190.7 ± 54.2	180.3 ± 51.6	190.4 ± 54.1	0.310
Platelet function tests (groups^a^, patient number, %)	ARU	835 (87.1):124 (12.9)	26 (89.7):3 (10.3)	861 (87.1):127 (12.9)	0.682
	PRU	896 (93.4):63 (6.6)	27 (93.1):2 (6.9)	923 (93.4):65 (6.9)	1.000
Blood tests	GPT (U/L)	24.1 ± 13.8	22.5 ± 15.8	24.1 ± 13.9	0.530
	GOT (U/L)	26.3 ± 12.9	28.3 ± 37	26.4 ± 14.1	0.465
	BUN (mg/dL)	16.4 ± 5.9	17.1 ± 6.7	16.4 ± 5.9	0.509
	Creatinine (mg/dL)	0.8 ± 0.3	0.9 ± 0.3	0.8 ± 0.3	0.079
	Homocysteine (µmol/L)	11.6 ± 5.1	14.0 ± 6.7	11.6 ± 5.1	0.014
	Fibrinogen (mg/dL)	306 ± 68.6	309 ± 81.9	306.1 ± 68.9	0.814
	Total cholesterol (mg/dL)	181.1 ± 43.6	188 ± 48.6	181.3 ± 43.8	0.401
	LDL (mg/dL)	109.2 ± 37.6	118.8 ± 41.2	109.5 ± 37.8	0.178
	HDL (mg/dL)	46.8 ± 12.8	47.1 ± 13.4	46.8 ± 12.8	0.905
	Triglyceride (mg/dL)	144 ± 118	118.6 ± 45.8	143.3 ± 116.5	0.248
	hsCRP (g/L)	2.5 ± 6.5	3.4 ± 7.5	2.5 ± 6.5	0.469
	White blood cells (/µL)	7.6 ± 2.3	8.2 ± 2.5	7.6 ± 2.3	0.188
	Hemoglobin (g/dL)	13.9 ± 1.7	14.6 ± 1.7	14 ± 1.7	0.025
	Platelets (10^3^/µL)	231.2 ± 59.3	233.1 ± 57.6	231.2 ± 59.2	0.865
	Hemoglobin A1c (%)	6.3 ± 1.3	6.9 ± 1.7	6.3 ± 1.3	0.009
Cyclooxygenase and lipoxygenase mediators	TXB2	40.7 ± 36.1	45.3 ± 31.9	40.9 ± 36	0.501
	PGE2	484 ± 635.2	638.3 ± 1162.3	488.5 ± 656.2	0.212
	6-keto PGF1α	9509 ± 17451.3	8859.1 ± 10,345	9490 ± 17281.5	0.842
	LTB4	803.2 ± 664.8	868.6 ± 588.2	805.1 ± 662.5	0.601
	LXA4	292.6 ± 343.3	213.2 ± 131.3	290.2 ± 339.2	0.215
Single nucleotide polymorphisms (patient number, %)	CYP2C19*2 (GG/GA/AA)	485 (50.6)/387 (40.4)/87 (9.1)	15 (51.7)/12 (41.4)/2 (6.9)	500 (50.6)/399 (40.4)/89 (9.0)	0.922
	CYP2C19*3 (GG/GA/AA)	779 (81.2)/174 (18.1)/6 (0.6)	21 (72.4)/8 (27.6)/0 (0.0)	800 (81.0)/182 (18.4)/6 (0.6)	0.404
	*COX1* (GG/GA/AA)	835 (87.1)/121 (12.6)/3 (0.3)	25 (86.2)/4 (13.8)/0 (0.0)	860 (87.0)/125 (12.7)/3 (0.3)	0.940
	*COX2* (CC/CG/GG)	854 (89.1)/102 (10.6)/3 (0.3)	26 (89.7)/3 (10.3)/0 (0.0)	880 (89.1)/105 (10.6)/3 (0.3)	0.954
	*ALOX5* (AA/AG/GG)	624 (65.1)/295 (30.8)/40 (4.2)	18 (62.1)/10 (34.5)/1 (3.4)	642 (65.0)/305 (30.9)/41 (4.1)	0.905
	*PGIS* (AA/AG/GG)	403 (42.0)/416 (43.4)/140 (14.6)	12 (41.4)/15 (51.7)/2 (6.9)	415 (42.0)/431 (43.6)/142 (14.4)	0.446
	*PGI*R (GG/GA/AA)	905 (94.4)/50 (5.2)/4 (0.4)	29 (100.0)/0 (0.0)/0 (0.0)	934 (94.5)/50 (5.1)/4 (0.4)	0.422
	*TXAS1* (CC/AC/AA)	491 (51.2)/389 (40.6)/79 (8.2)	9 (31.0)/14 (48.3)/6 (20.7)	500 (50.6)/403 (40.8)/85 (8.6)	0.021
	*TXA2R (*GG/GA/AA)	153 (16.0)/442 (46.1)/364 (38.0)	1 (3.4)/16 (55.2)/12 (41.4)	154 (15.6)/458 (46.4)/376 (38.1)	0.182
Promoter methylations (%)	*COX1*	2.6 ± 1.8	3.1 ± 1.9	2.7 ± 1.8	0.162
	*COX2*	3.6 ± 2.8	4.0 ± 2.7	3.6 ± 2.7	0.421
	*ALOX5*	2.9 ± 2.9	2.9 ± 3.2	2.9 ± 2.9	0.931
	*ALOX12*	31.2 ± 10.4	34.7 ± 7.8	31.3 ± 10.4	0.076
	*ALOX15*	5.0 ± 2.6	4.9 ± 1.9	5.0 ± 2.6	0.791
	*PGE2S*	0.5 ± 1.7	0.7 ± 1.5	0.5 ± 1.7	0.611
	*PGE2R*	0.2 ± 0.9	0.1 ± 0.5	0.2 ± 0.9	0.534
	*PGIS*	2.9 ± 2.9	3.1 ± 4.1	2.9 ± 3.0	0.765
	*PGIR*	89.4 ± 2.8	89.4 ± 1.6	89.4 ± 2.8	0.870
	*TXAS1*	1.6 ± 2.3	1.9 ± 2.4	1.6 ± 2.3	0.447
	*TXA2R*	9.0 ± 3.3	8.6 ± 3.2	9.0 ± 3.3	0.524

*6-keto* PGF1α, *6-keto* Prostaglandin F1α, *ALOX5* 5-lipoxygenase gene, *ALOX12* 12-lipoxygenase gene, *ALOX15* 15-lipoxygenase gene, *ARU* Average of aspirin reaction units measured 5 days and 12 weeks after aspirin and clopidogrel administration, *BUN* Blood urea nitrogen, *COX1* Cyclooxygenase 1 gene, *COX2* Cyclooxygenase 2 gene, *CYP2C19* Cytochrome P450 2C19 gene, *GOT* Glutamic oxaloacetic transaminase, *GPT* Glutamic pyruvate transaminase, *HDL* High-density lipoprotein, *hsCRP* High-sensitivity C-reactive protein, *LDL* Low-density lipoprotein, *LTB4* Leukotriene B4, *LXA4* Lipoxin A4, *PGE2* Prostaglandin E2, *PGE2S* Prostaglandin E2 synthase gene, *PGE2R* Prostaglandin E2 receptor gene, *PGIS* Prostaglandin I synthase gene, *PGIR* Prostaglandin I receptor gene, *PRU* Average of P2Y12 reaction units measured 5 days and 12 weeks after aspirin and clopidogrel administration, *TXAS1* Thromboxane A synthase 1 gene, *TXA2R* Thromboxane A2 receptor gene, *TXB2* Thromboxane B2

^a^Inhibition and non-inhibition groups classified based on the reference range of ≤ 550 ARU or ≤ 270 PRU

### Evaluation of clinical and laboratory resistance to aspirin and clopidogrel

Patients were administered 300 mg aspirin and 300 mg clopidogrel after initial diagnosis of ischemic stroke and subsequently received 100 mg aspirin and 75 mg clopidogrel daily for 12 weeks, then, we assessed clinical and laboratory resistance to aspirin and clopidogrel. Clinical resistance was evaluated as recurrence of TIA or ischemic stroke during the 12-week follow-up. Laboratory resistance was evaluated based on the aspirin reaction unit (ARU) for aspirin and P2Y12 reaction unit (PRU) for clopidogrel using VerifyNow (Werfen, Barcelona, Spain) 5 days and 12 weeks following administration of the two drugs. We used average of ARU and PRU obtained from different time intervals as the representative antiplatelet functions occurred after aspirin and clopidogrel administrations for individual patients. To test independent variables related to laboratory resistance of aspirin and clopidogrel, we initially tried to compare between inhibition and non-inhibition groups classified by the reference range of ≤ 550 ARU according to the VerifyNow instruction and ≤ 270 PRU recommended for East Asians in a previous study [14], and next, used ARU and PRU levels themselves measured from the individual patients.

### Blood collection

To test mediators, SNPs, and promoter methylation of the target genes, we collected 3 mL whole blood in a citrate-coated tube concurrently with the blood collection for the first ARU and PRU tests. Plasma and buffy coat were separated from each tube after centrifugation for 15 min at 100 × *g* and stored at -80 °C until next use. DNA was extracted from the buffy coats using the DNeasy Blood & Tissue Kit (Cat. no. 69,506, Qiagen, Valencia, CA, USA) and stored at -20 °C until further SNP and promoter methylation evaluation.

### Measurement of target mediators, genetic and epigenetic characteristics of the target genes

**Fig. 1:**
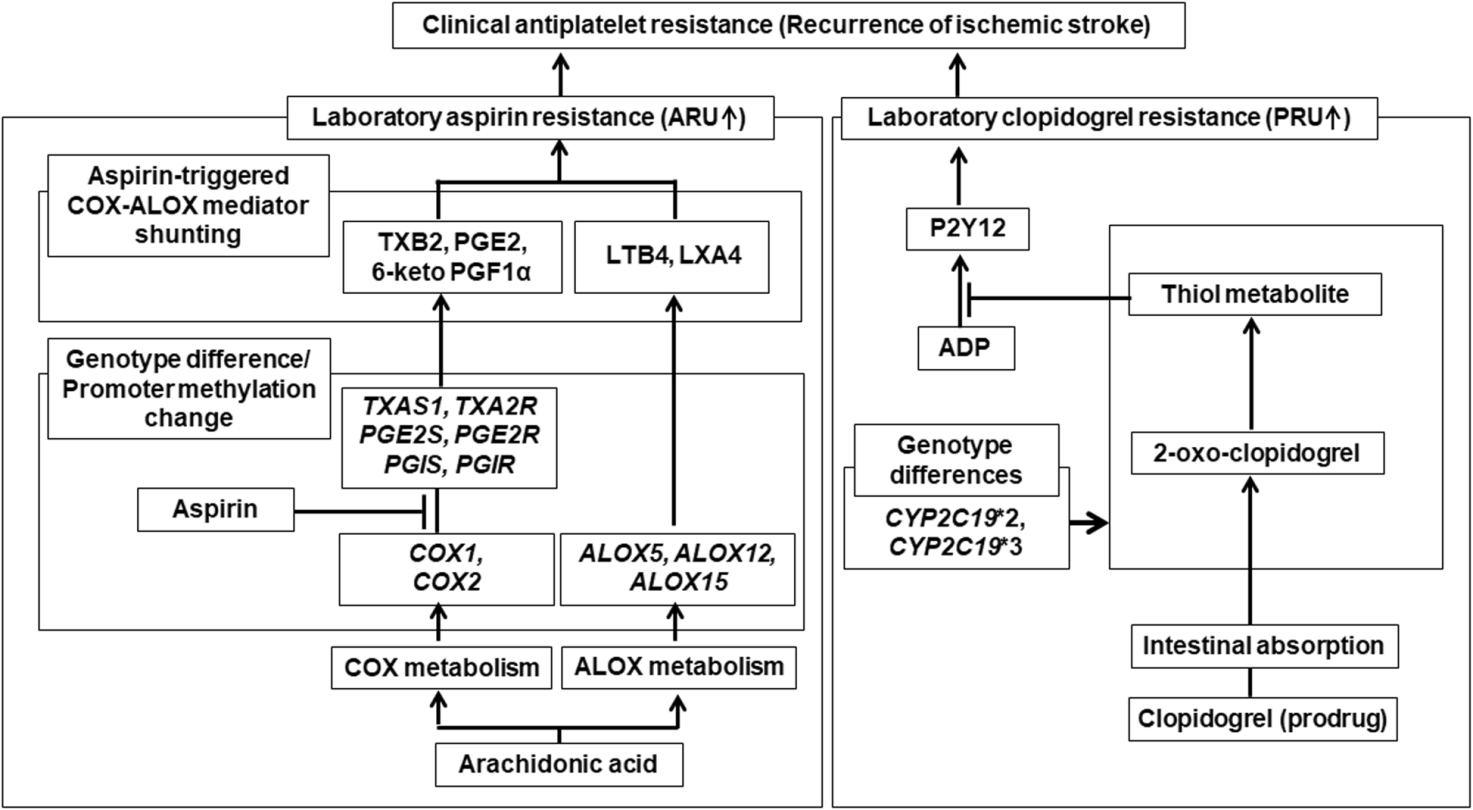
Mediators and genes of arachidonic acid metabolism and clopidogrel activation targeted in the present study [4, 10]. 6-keto PGF1α, 6-keto prostaglandin F1α; ADP, adenosine diphosphate; *ALOX5*, 5-lipoxygenase gene; *ALOX12*, 12-lipoxygenase gene; *ALOX15*, 15-lipoxygenase gene; ARU, aspirin reaction units; *COX1*, cyclooxygenase 1 gene; *COX2*, cyclooxygenase 2 gene; *CYP2C19*, cytochrome P450 2C19 gene; LTB4, leukotriene B4; LXA4, lipoxin A4; P2Y12, a chemoreceptor for adenosine diphosphate; PGE2, prostagladin E2; *PGE2S*, prostaglandin E2 synthase gene; *PGE2R*, prostaglandin E2 receptor gene; *PGIS*, prostaglandin I synthase gene; *PGIR*, prostaglandin I receptor gene; PRU, P2Y12 reaction units; *TXAS1*, thromboxane A synthase 1 gene; *TXA2R*, thromboxane A2 receptor gene; TXB2, thromboxane B2

To evaluate mediators, genomic and epigenomic characteristics, we selected mediators and genes related with the arachidonic acid metabolisms, clopidogrel activation and/or cardiovascular diseases described in the previous studies. To test differences of the mediator levels in plasma, we targeted thromboxane A2 (TXA2), prostaglandin E2 (PGE2), and prostaglandin I2 (PGI2) for the COX pathway, and, leukotriene B4 (LTB4) and lipoxin A4 (LXA4) levels for the ALOX pathway (Fig. 1) [4, 7]. TXA2 and PGI2 are labile and difficult to measure from human body fluid [15]. Therefore, we measured the levels of their stable metabolites thromboxane B2 (TXB2) of TXA2 and 6-keto prostaglandin F1α (6-keto PGF1α) of PGI2. Plasma levels of PGE2 (cat. no.: MBS3801155, MyBioSource, San Diego, CA, USA), TXB2 (cat. no.: MBS2600558, MyBioSource), 6-keto PGF1α (cat. no.: ADI-901-004, Enzo Life Sciences, Bruxelles, Belgium), LTB4 (cat. no.: ADI-901-068, Enzo Life Sciences), and LXA4 (cat. no.: MBS160535, MyBioSource) were measured using competitive or sandwich type enzyme-linked immunosorbent assay (ELISA) according to the manufacturers’ instructions. We duplicated ELISA tests for the individual mediators, and used average of these duplicates as the representative level of each target mediator.

To evaluate genomic characteristics, we selected 11 SNPs, namely *COX1* (rs3842788) A > G [16], *COX2* (rs20417) G > C [17, 18], thromboxane A synthase 1 (*TXAS1*, rs41708) A > C [17, 18], thromboxane A2 receptor (*TXA2R*, rs1131882) G > A [18], prostaglandin I synthase (*PGIS*, rs5602) G > A [17] and receptor (*PGIR*, rs4987262) A > G [19], *ALOX5* (rs745986) G > A [20], *ALOX12* (rs1126667) G > A [21], and *ALOX15* (rs34210653) A > G [22], participating in COX and ALOX metabolism, as well as CYP2C19*2 (rs4244285) A > G and CYP2C19*3 (rs4986893) A > G [23] involved in the hepatic activation of clopidogrel (Fig. 1). Prostaglandin E2 synthase (*PGE2S*) and receptor (*PGE2R*) were not included for the SNP tests because no clear relationship has been described between specific SNPs of the two genes and cardiovascular disease. Genotypes of the 11 SNPs were evaluated using SNP-Genotyping pyrosequencing using primers designed for each SNP (Supplementary Table 1). We performed duplex (CYP2C19*2-*3) or triplex (*COX2-COX1-ALOX5*, *ALOX12-TXA2R-PGIR*, *TXAS1-ALOX15-PGIS*) SNP-Genotyping-pyrosequencing for the two or three individual genes (Supplementary Table 2). Each duplex or triplex PCR was performed in 20 µL of a premix PCR kit (AccuPower [®] PyroHotStart Taq PCR PreMix, cat. no. K-2611, Bioneer, Daejeon, South Korea), to which we added 15 ng of DNA and 0.1 mmol/L of forward and reverse primers for two or three genes (Supplementary Table 1). The PCR protocol was as follows: 45 PCR cycles (30 s at 95 °C for denaturation, 30 s at annealing temperature for duplex or triplex PCR [Supplementary Table 2], and 30 s at 72 °C for extension). Subsequently, the 15 µL duplex or triplex PCR product was mixed with streptavidin-coated Sepharose beads (GE Healthcare, Chicago, IL, USA) in PyroMark binding buffer (Qiagen). The DNA-coated beads were denatured into single-stranded DNA template beads and released into a well of the PyroMark 96-well plate containing 40 µL PyroMark annealing buffer (Qiagen). After adding 0.2 µM sequencing primers for the two or three individual genes into each well, genotypes of the two or three genes (Supplementary Table 2) of each PCR product were sequenced using a pyrosequencing machine (PyroMark Q96 ID, Qiagen).

To evaluate epigenomic characteristics, we tested the promoter methylation levels of *COX1*, *COX2*, *PGE2S*, *PGE2R*, *PGIS*, *PGIR*, *TXAS1*, and *TXA2R* for the COX pathway, as well as *ALOX*5, *ALOX12*, and *ALOX15* for the ALOX pathway (Fig. 1). The evaluated genes had promoter CpG islands defined by > 0.5 GC percentage and > 0.6 observed/expected CpG ratio (Supplementary Fig. 1). *CYP2C19* *2 and *3, which has no promoter CpG island, was not targeted for the methylation evaluation. Promoter methylation levels were evaluated using DNA-Methylation pyrosequencing using primers designed for individual genes (Supplementary Table 3). The PCR protocol was as follows: 45 PCR cycles (30 s at 95 °C for denaturation, 30 s at annealing temperature for individual genes [Supplementary Table 3], and 30 s at 72 °C for extension). Then, single-stranded DNA template beads for each PCR product were prepared and transferred into a PyroMark 96-well plate, similar to the process used for SNP genotype pyrosequencing. After adding 0.4 µM sequencing primer into each well, the methylation status of each PCR product was obtained using the pyrosequencing machine. The methylation level of each gene was represented as an average value from all pyrosequenced CpG sites of the gene.

### Statistical analyses

Before including the SNP data of the 11 target genes for the total dataset, we determined the Hardy–Weinberg equilibrium and allele frequency of each SNP. Here, we only included SNPs satisfying a p-value > 0.01 for both analyses [24, 25]. After checking the completeness of the dataset, we imputed the missing values to prepare the final total dataset using an imputation algorithm package (missCompare) developed for R (ver. 4.1.3) as previously described (https://github.com/Tirgit/missCompare).

We first identified variables related to clinical resistance in the final dataset. We compared the differences in the platelet function test results, cardiovascular risk factors, blood test profiles, mediator levels, SNP genotypes, and promoter methylations of the target genes between the recurrence and non-recurrence groups. An independent t-test was used for continuous variables and a chi-squared test was used for categorical variables. We subsequently performed hierarchical logistic regression analysis to evaluate whether the recurrence prediction model estimated with only cardiovascular risk factors and blood tests could enhance performance, including accuracy, specificity, sensitivity, and area under (AUC) the receiver operating characteristic curve (ROC). This was achieved by adding the mediators, SNPs, and promoter methylation data to these data. We calculated Youden’s index from the ROC data of the logistic regression model fitted with all variables and selected a classification threshold close to the maximum Youden’s index. We performed Hosmer-Lemeshow goodness-of-fit test to evaluate whether the probability of success of the logistic regression models is close to the true probability.

We identified variables related to the laboratory resistance to aspirin and clopidogrel measured using ARU and PRU. We initially classified the patients into inhibition and non-inhibition groups based on the inhibition criteria of ≤ 550 ARU and ≤ 270 PRU. The patient number in the ARU and PRU inhibition group was markedly imbalanced by the inhibition reference criteria. Therefore, we used the ARU and PRU levels of individual patients to evaluate independent variables related to laboratory resistance to aspirin and clopidogrel. We analyzed the relationships between ARU and PRU and all tested variables using correlation analysis and independent t-test or analysis of variance (ANOVA) tests. Subsequently, we performed hierarchical linear regression analysis to evaluate whether the ARU and PRU prediction model could enhance their performance by adding the mediator, SNP, and promoter methylation data to the cardiovascular risk factors and blood tests. All linear regression analyses used dummy variables for the categorical variables. The inclusion criteria for the reference group for dummy coding was as follows: men, a previous history of hypertension, diabetes, and smoking. The dummy variables for the individual target SNPs were wild genotypes of CYP2C19*2 G/G, CYP2C19*3 G/G, *COX1* G/G, *COX2* C/C, *ALOX5* A/A, *PGIS* A/A, *PGIR* G/G, *TXAS1* C/C, and *TXA2R* G/G. To evaluate whether the variables selected for the linear ARU and PRU prediction linear models is statistically significant, we used p-value of ANOVA results obtained from the linear regression analysis.

The univariate comparison, Youden’s index calculation, logistic and linear regression analysis, and dummy coding were performed using SPSS software (ver. 26.0, IBM Inc., Chicago, IL, USA). Statistical significance was set at *P* < 0.05.

## Results

### Preparation of the final dataset

This STRAPER study initially enrolled 1,002 patients from six hospitals after screening. However, five patients deviated from the study protocol due to incomplete compliance during their follow-up periods and nine did not satisfy the criteria for the platelet function tests, resulting in 988 study participants. Of the 11 target SNPs, *ALOX15* (rs34210653) showed only a G allele and *ALOX12* (rs1126667) had a p-value < 0.001 on the exact test of the Hardy–Weinberg equilibrium analysis (Supplementary Table 4). We included the remaining nine SNPs, namely *ALOX5* (rs745986), *COX1* (rs3842788), *COX2* (rs20417), CYP2C19*2 (rs4244285) and *3 (rs4986893), *PGIS* (rs5602), *PGIR* (rs4987262), *TXAS1* (rs41708), and *TXA2R* (rs1131882) for the total dataset. We prepared a complete final dataset comprising the 48 variables (recurrence of ischemic stroke, five risk factors, two platelet function tests, 15 blood tests, five mediators, nine SNPs, and 11 promoter methylations; Table 1) of 988 patients after the imputation of 806 missing values (0.17%) among the 47,424 total values (Supplementary Table 5).

### Identification of variables related to prediction of ischemic stroke recurrence

Clinical resistance characterized by TIA or ischemic stroke recurrence was observed in 29 (2.9%, 65.9 ± 9.1 years, men: women = 24:5) of the 988 patients (68.9 ± 9.3 years, men: women = 644:344) over the 12 weeks of aspirin and clopidogrel administration. Platelet function tests, ARU, PRU, and the five mediators were not different between the two groups (Table 1). Patients with recurrence were younger (*p* = 0.071), predominantly men (*p* = 0.044) and had a smoking history (*p* < 0.001). Furthermore, they had higher levels of homocysteine (*p* = 0.014), hemoglobin (*p* = 0.025), hemoglobin A1c (HbA1c, *p* = 0.009), and creatinine (*p* = 0.079), and more frequently had the *TXAS1* (rs41708) A/A genotype (*p* = 0.021) and higher *ALOX12* methylation (*p* = 0.076) than those in the non-recurrence group (Table 1).

In the hierarchical logistic regression analysis, *TXAS1* (rs41708) A/A genotype and *ALOX12* promoter methylation were added to the cardiovascular risk factors of smoking history and the triglyceride and HbA1c test results as independent variables for the recurrence prediction model (AUC, 0.863 [confidence interval = 0.811–0.915], *p* < 0.001) (Table 2). We used a classification threshold of 0.3, which is close to the maximum Youden’s index of 0.34, to evaluate the performance of the logistic model fitted for the 3% recurrence of the present dataset. Sensitivity to predict recurrence was 3.4% (accuracy, 96.7%; specificity, 99.5%) of the logistic model fitted only with the cardiovascular risks and blood tests. This was enhanced to 13.4% (accuracy, 96.7%; specificity, 99.2%) after adding the mediators and genomic and epigenomic variables (Table 2). Hosmer-Lemeshow goodness-of-fit test showed higher > 0.05 p-vale in the regress model performed with the cardiovascular risks and blood tests (*p* = 0.875) and in the model performed after adding mediators, genomic and epigenomic variables to the risks (*p* = 0.706). Greater 0.05 p-value of the Hosmer-Lemeshow tests obtained before and after adding the mediators, genomic and epigenomic variables indicated that the logistic regression models are acceptable and are considered good fits.

**Table 2:** Hierarchical logistic regression analysis to predict clinical resistance

Variable groups and model performance	Variables	Model 1		Model 2	
		B	Exp(B)	B	Exp(B)
Constant		-9.833	0.000***	-13.085	0.000***
Risk factors/blood tests	Smoking (Yes)	1.198	3.313**	1.214	3.368 **
	Triglyceride	-0.008	0.992*	-0.008	0.992 *
	Hemoglobin A1c	0.262	1.300*	0.271	1.311*
Single nucleotide polymorphisms	*TXAS1* (rs41708) A/A			1.495	4.459*
Promoter methylation	*ALOX12*			0.037	1.038*
Model performance†	Specificity	99.5		99.2	
	Sensitivity	3.4		13.8	
	Accuracy	96.7		96.7	

†Classification threshold to test the model performance of each logistic regression model: 0.3

Model 1 = Regression analysis performed with variables including cardiovascular risk factors + blood tests

Model 2 = Regression analysis with variables including cardiovascular risk factors + blood tests + mediators + single nucleotide polymorphisms + promoter methylation

*B* Unstandardized coefficient, *β *Standardized coefficient, *p < 0.05; **p < 0.01; ***p < 0.001

Reference group for *TXAS1* (rs41708) = C/C

*ALOX12* 12-lipoxygenase gene, *TXAS1* Thromboxane synthase 1 gene

### Limitations while classifying patients into the inhibition and non-inhibition groups using the reference ranges of ARU and PRU

We initially classified all patients into inhibition and non-inhibition groups using the inhibition reference range ≤ 550 ARU and ≤ 270 PRU to evaluate aspirin and clopidogrel resistance. Of the total patients, 87% (861 patients) were classified into the ARU inhibition group and 93% (923 patients) into the PRU inhibition group (Table 1). The large imbalance between patient numbers of the inhibition and non-inhibition groups classified using the ARU and PRU reference range could lead to a bias in the statistical estimates and cause overfitting of the regression model during the identification of independent variables for laboratory resistance [26]. Thus, we performed linear regression analysis to predict ARU and PRU levels and subsequently identify independent variables related to laboratory resistance against aspirin and clopidogrel.

### Identification of variables related to ARU prediction

ARU was positively correlated with age and *COX1*, *PGE2S*, *PGE2R* and *TXAS1* promoter methylation. However, it was negatively correlated with total cholesterol, low density lipoprotein cholesterol, white blood cells, platelet, and LTB4 levels (Supplementary Table 6). No significant difference was observed between ARU and cardiovascular risk factors or genotypes of the target SNPs (Supplementary Table 6).

In the hierarchical linear regression analysis, LTB4 levels, *COX2* (rs20417) C/G and thromboxane A2 receptor (*TXA2R*, rs1131882) A/A genotypes, as well as *COX1* and *ALOX15* promoter methylation were added to platelet levels from the blood tests as independent variables for the ARU prediction model (Table 3). The performance of the ARU prediction model fitted with only clinical and blood test risk factors (*r* = 0.163, R2 = 0.027, adjusted R^2^ = 0.008, F = 1.396, *p* > 0.1) was enhanced after adding mediators and the genetic and epigenetic variables (*r* = 0.291, R2 = 0.084, adjusted R^2^ = 0.033, F = 1.626, *p* < 0.01) (Table 3). The linear regression model performed with only cardiovascular risks and blood tests showed > 0.1 p-value of ANOVA tests. However, after adding the mediators, genomic and epigenomic variables to the risk factor variables, the logistic model showed < 0.01 p-value of ANOVA, even though R2 and adjusted R2 of the model was broad, yet.

**Table 3:** Hierarchical linear regression analysis to predict ARU level

Variable groups and model performance		Model 1				Model 2			
		B	β	t value	VIF	B	β	t value	VIF
	Constant	509.475		20.138***		496.985		7.431***	
Blood tests	Platelet	-0.084	-0.087	-2.470*	1.247	-0.072	-0.075	-2.086**	1.305
Mediators	LTB4					-0.008	-0.089	-2.604**	1.191
Single nucleotide polymorphisms	*COX2* (rs20417) C/G					-12.344	-0.067	-2.085*	1.041
	*TXA2R* (rs1131882) A/A					-12.599	-0.107	-2.281*	2.249
Promoter methylation	*COX1*					2.73	0.086	2.471*	1.231
	*ALOX15*					-1.672	-0.076	-2.369*	1.063
Model summary	r	0.163				0.291			
	R^2^	0.027				0.084			
	Adjusted R^2^	0.008				0.033			
ANOVA	F value	1.396				1.626**			

Model 1 = Regression analysis performed with variables including cardiovascular risk factors + blood tests

Model 2 = Regression analysis with variables including cardiovascular risk factors + blood tests + mediators + single nucleotide polymorphisms + promoter methylations

*B *Unstandardized coefficient, *β *Standardized coefficient, *p < 0.05; **p < 0.01; ***p < 0.001

Reference group to create dummy variables: *COX2* (rs20417) = C/C; *TXA2R* (rs1131882) = G/G.

*ANOVA* Analysis of variance, *VIF* Variance inflation factor

*ALOX15 *15-lipoxygenase gene, *COX1* Cyclooxygenase 1, *COX2 C*cyclooxygenase 2 gene, *LTB4* Leukotriene B4, *TXA2R* Thromboxane A2 receptor gene

### Identification of the variables related to PRU prediction

PRU was positively correlated with age and blood urea nitrogen and negatively correlated with glutamic pyruvate transaminase, total cholesterol, low density lipoprotein cholesterol, and hemoglobin and platelet levels. However, it had no significant correlation with the target promoter methylation (Supplementary Table 6). Moreover, the PRU level was high in women and smokers (Supplementary Table 6). The genotypes CYP2C19*2 (rs4244285) A/A and G/A, CYP2C19*3 (rs4986893) A/A and G/A, and *PGIR* (rs4987262) G/A showed higher PRU than the other genotypes of each gene (Supplementary Table 6).

In the hierarchical linear regression analysis, CYP2C19*2 (rs4244285) A/A and G/A, CYP2C19*3 (rs4986893) A/A and G/A, and *PGIR* (rs4987262) G/A genotypes and *COX2* and *TXAS1* promoter methylation were added to sex, age, as well as creatinine and platelet counts as independent variables for the PRU prediction model (Table 4). The performance of the PRU prediction model fitted with only the cardiovascular risks and the blood tests (*r* = 0.293, R2 = 0.086, adjusted R^2^ = 0.068, F = 4.779, *p* < 0.01) was enhanced after adding the molecular genomic and epigenomic variables (*r* = 0.503, R2 = 0.253, adjusted R^2^ = 0.210, F = 5.959, *p* < 0.001) (Table 4). Before and after adding mediators, genomic and epigenomic variables to the risk factors, p-value of ANOVA showed < 0.05, and, R2 and adjusted R2 was enhanced after adding of the mediators, genomic and epigenomic variables to the cardiovascular risk factors.

**Table 4:** Hierarchical linear regression analysis to predict PRU level

Variable groups and model performance	Variables	Model 1				Model 2			
		B	β	t value	VIF	B	β	t value	VIF
	Constant	172.136		7.413***		94.755		1.656†	
Risk factors/blood tests	Sex (women)	25.070	0.221	5.797***	1.536	24.392	0.215	6.004***	1.600
	Age	0.650	0.111	3.105**	1.365	0.791	0.136	4.010***	1.431
	Creatinine	19.173	0.098	2.455*	1.685	15.468	0.079	2.088*	1.790
	Platelet	-0.060	-0.066	-1.916†	1.247	-0.078	-0.085	-2.629**	1.305
Single nucleotide polymorphisms	CYP2C19*2 (rs4244285) A/A					56.781	0.301	9.661***	1.210
	CYP2C19*2 (rs4244285) G/A					29.197	0.265	8.801***	1.132
	CYP2C19*3 (rs4986893) A/A					70.87	0.102	3.486***	1.066
	CYP2C19*3 (rs4986893) G/A					30.385	0.218	7.348***	1.098
	*PGIR* (rs4987262) G/A					20.862	0.085	2.907**	1.057
Promoter methylation	*COX2*					-1.597	-0.081	-2.206*	1.691
	*TXAS1*					1.771	0.075	2.308*	1.333
Model summary	r	0.293				0.503			
	R^2^	0.086				0.253			
	Adjusted R^2^	0.068				0.210			
ANOVA	F value	4.779***				5.959***			

Model 1 = Regression analysis performed with variables including cardiovascular risk factors + blood tests

Model 2 = Regression analysis with variables including cardiovascular risk factors + blood tests + mediators + single nucleotide polymorphisms + promoter methylation

*B *Unstandardized coefficient, *β *Standardized coefficient, †< 0.1; *p < 0.05; **p < 0.01; ***p < 0.001

Reference group to create dummy variables: Sex = men; CYP2C19*2 (rs4244285) = G/G; CYP2C19*3 (rs4986893) = G/G; *PGIR* (rs4987262) = G/G.

*ANOVA* Analysis of variance, *VIF* Variance inflation factor

*COX2* Cyclooxygenase 2 gene, *CYP2C19 *Cytochrome p450 2C19, *PGIR *Prostaglandin I receptor gene, *TXAS1 *Thromboxane A synthase 1 gene

## Discussion

The present study revealed that the prediction of clinical and laboratory resistance to aspirin and clopidogrel could enhance by adding mediators, genomic and epigenomic characteristics of the genes participating in pharmacodynamic COX and ALOX metabolism of arachidonic acid and in pharmacokinetic activation of clopidogrel to the established cardiovascular risk factors. Although we did not include all genes participating in arachidonic acid metabolism and clopidogrel activation, we showed that different mediators, SNPs, and promoter methylations targeted in the present study could be molecular genomic and epigenomic markers to predict the clinical and laboratory resistance to aspirin and clopidogrel.

The regression model for predicting clinical resistance included the *TXAS1* (rs41708) A/A genotype and *ALOX12* promoter methylation as independent variables, in addition to smoking history, triglyceride, and HbA1c levels, which are established cardiovascular risk factors. The *TXAS1* (rs41708) SNP [17, 18] and *ALOX12* promoter methylation status [27] have been reported as genomic and epigenomic markers related to ischemic strokes and atherosclerosis. The present logistic model was fitted for data of the patient population with a 3% incidence of clinical resistance within 12 weeks of aspirin and clopidogrel administration. After application of the 0.3 classification threshold for the logistic model, sensitivity to predict clinical resistance was enhanced from 3.4% before to 13.4% after adding the mediators, genomic and epigenomic variables to the established risk factors. However, the markedly imbalanced incidence of clinical resistance could have caused the biased estimation and overfitting risks to the clinical resistance prediction model [26]. Therefore, future studies with more patients and a follow-up period of more than 12 weeks are required to verify the significance of the genomic and epigenomic variables in clinical resistance prediction.

We initially classified the inhibition group based on the recommended reference range of ≤ 550 ARU and ≤ 270 PRU to evaluate laboratory resistance. However, 87% of the study population showed an ARU ≤ 550, and, 93% showed a PRU ≤ 270. Previous studies have also revealed an ARU of ≤ 550 in 95% of patients [28] or have used a PRU of ≤ 208 [29], ≤230 [30] or about ≤ 270 [14] to define the clopidogrel inhibition group instead of the recommended range by the instruction manual. The considerable imbalance between patient numbers of inhibition and non-inhibition groups classified using the ≤ 550 ARU and the ≤ 270 PRU reference could have also caused the overfitting problem [26] during the development of the logistic model for predicting aspirin laboratory resistance, similar to the clinical resistance prediction analysis. Therefore, gold standard methods are required to evaluate platelet function and the reference range to define platelet function inhibition against aspirin and clopidogrel.

We performed linear regression modeling to predict ARU and PRU levels to evaluate variables related to laboratory resistance. The linear ARU prediction model included *COX2* (rs20417) C/G and *TXA2R* (rs1131882) A/A genotypes, *COX1* and *ALOX15* promoter methylation, and LTB4 levels as independent variables. These were added to the platelet levels included in the blood tests. *COX2* (rs20417) C/G [17, 18] and *TXA2R* (rs1131882) A/A [18] genotypes were identified as genomic characteristics related to aspirin resistance. The characteristics of the *COX1* (rs3842788) [8] and *ALOX15* genotypes [31] were related to aspirin resistance, even though genotypes of the two genes were not included as variables for the present ARU prediction model. Moreover, the epigenomic characteristics of the genes were related to laboratory resistance to aspirin when *COX1* and *ALOX15* promoter methylation were added as variables to the ARU prediction.

LTB4, an ALOX mediator, showed an inverse association with the ARU prediction model. Aspirin-triggered COX–ALOX shunting increases cysteinyl leukotriene levels in aspirin-exacerbated respiratory disease [32, 33]. The aspirin-triggered LTB4 changes could activate peripheral monocytes [34] and interaction between leukocytes and endothelial cells in atherogenesis [35] as well as in aspirin-exacerbated respiratory disease [32]. The inverse association between LTB4 and the ARU prediction model in the present study showed another pharmacodynamic evidence of aspirin-triggered COX–ALOX shunting, i.e., COX1 inhibition after aspirin administration.

The linear PRU prediction model included CYP2C19*2 (rs4244285) A/A and A/G, CYP2C19*3 (rs4986893) A/A and A/G, and *PGIR* (rs4987262) G/A genotypes, as well as *COX2* and *TXAS1* promoter methylation as independent variables in addition to sex, age, creatinine and platelet count. The loss-of-function genotypes CYP2C19*2 (rs4244285) A/A and A/G and CYP2C19*3 (rs4986893) A/A and A/G have been reported as genomic characteristics related to clopidogrel resistance [36, 37]. The genotypic characteristics of CYP2C19*2 (rs4244285) and CYP2C19*3 (rs4986893) as independent variables for the PRU prediction model in the present study supported the previously identified pharmacokinetic importance of *CYP2C19* [1] related to clopidogrel resistance. However, the relationships between the *PGIR* (rs4987262) SNP and *COX2* and *TXAS1* methylation changes and PRU changes warrant further study.

This study had some limitations. First, gold-standard methods are required to measure platelet function after antiplatelet agent administration. Several platelet function tests, including VerifyNow, have been introduced to measure platelet function without consistency between the test results [38]. Therefore, reference ranges recommended by gold-standard tests are required to define an inhibition group based on platelet function. Second, more target pathways and genes involved in arachidonic acid metabolism and clopidogrel activation are needed to identify molecular genomic and epigenomic markers related to aspirin and clopidogrel resistance. The genes converting hydroxyeicosatetraenoic acid into leukotrienes [39] and participating in the absorption and hydrolysis of clopidogrel [7] must be extensively evaluated to elucidate the pharmacodynamic and pharmacokinetic characteristics of aspirin and clopidogrel resistance. Finally, further investigations are required to verify the significance of the present markers from the different populations by enrolling enough patients and considering differences in ethnicity [40] and exposure to varying environments [10, 11]. 

## Conclusions

The present multi-center prospective cohort study identified different mediators, as well as genomic and epigenomic characteristics of arachidonic acid metabolism and hepatic clopidogrel activation as independent markers for predicting clinical and laboratory resistance to aspirin and clopidogrel.

### Supplementary Information

Supplementary Material 1.

Supplementary Material 2.

## Data Availability

No datasets were generated or analysed during the current study.

## Abbreviations

**ALOX**: Arachidonate lipoxygenase
**ARU**: Aspirin reaction unit
**AUC**: Area under the receiver operating characteristic curve
**COX**: Cyclooxygenase
***CYP2C19***: Cytochrome P450 2C19 gene
**ELISA**: Enzyme-linked immunosorbent assay
**HbA1c**: Hemoglobin A1c
**IRB**: Institutional Review Board
**LTB4**: Leukotriene B4
**LXA4**: Lipoxin A4
**PGE2**: Prostaglandin E2
***PGE2R***: Prostaglandin E2 receptor gene
***PGE2S***: Prostaglandin E2 synthase gene
**PGI2**: Prostaglandin I2
***PGIR***: Prostaglandin I receptor gene
***PGIS***: Prostaglandin I synthase gene
**PRU**: P2Y12 reaction unit
**ROC**: Receiver operating characteristic
**SNP**: Single nucleotide polymorphism
**STRAPER study**: A prospective cohort clinical trial titled ‘Multi-center, prospective, cohort study to evaluate the relationship of STroke Recurrence and Anti-PlatElet Resistance in ischemic stroke patients’
**TIA**: Transient ischemic attack
**TXA2**: Thromboxane A2
***TXA2R***: Thromboxane A2 receptor gene
***TXAS1***: Thromboxane A synthase 1 gene
