# Supplementary material for: Molecular genomic and epigenomic characteristics related to aspirin and clopidogrel resistance
Source: BMC Med Genomics. 2024 Jun 20;17:166. doi: 10.1186/s12920-024-01936-1 (PMC11188263; doi:10.1186/s12920-024-01936-1)

**Supplemental Material**

**Supplementary table 1. Primer sequences for single nucleotide genotyping for pyrosequencing of the target genes**

| Gene | Reference SNP number | Nucleotide variation | Primers | Primer sequence (5' -> 3') | PCR product (bp) |
| --- | --- | --- | --- | --- | --- |
|  |  |  |  |  |  |
| *CYP2C19**2 | rs4244285 | G>A | Forward | (B)-TGCAATAATTTTCCCACTATCATTG | 152 |
|  |  |  | Reverse | CAATCAATAAAGTCCCGAGGGT |  |
|  |  |  | Sequencing | TTAAGTAATTTGTTATGGGT |  |
|  |  |  | Analyzing seq. (Reverse C>T) | TCC*C/T*GGGAAATAATCAATGATAGTGGGAA |  |
| *CYP2C19**3 | rs4986893 | G>A | Forward | (B)-TCCCTGCAATGTGATCTGCT | 217 |
|  |  |  | Reverse | ATGTACTTCAGGGCTTGGTCAAT |  |
|  |  |  | Sequencing | AACTTGGCCTTACCTGG |  |
|  |  |  | Analyzing seq. (Reverse C>T) | AT*C/T*CAGGGGGTGCTTACAATCCTGATGT |  |
| *COX1* | rs38427881 | G>A | Forward | CATTTCTCACCCACAGTGAATCC | 72 |
|  |  |  | Reverse | (B)-GCCGAAGCGGACACAGAT |  |
|  |  |  | Sequencing | GTTGTTACTATCCATGCC |  |
|  |  |  | Analyzing seq. (Forward G>A) | A*G/A*CACCAGGGCATCTGTGTCCGCTTC |  |
| *COX2* | rs20417 | C>G | Forward | (B)-GTACCTTCACCCCCTCCTTGT | 111 |
|  |  |  | Reverse | CCGTGGAGCTCACATTAACTATTT |  |
|  |  |  | Sequencing | GGAGAATTTACCTTTCCC |  |
|  |  |  | Analyzing seq. (Reverse G>C) | *G/C*CCTCTCTTTCCAAGAAACAAGGAGG |  |
| *ALOX5* | rs745986 | A>G | Forward | (B)-GTACCTTCACCCCCTCCTTGT | 92 |
|  |  |  | Reverse | CCGTGGAGCTCACATTAACTATTT |  |
|  |  |  | Sequencing | AGGTTTAAACTGAGAGCA |  |
|  |  |  | Analyzing seq. (Reverse T>C) | AGC*T/C*GAAGGGTTATGACTCGAA |  |
| *ALOX12* | rs1126667 | A>G | Forward | (B)-CCTCGGGGATGGAAGAGC | 177 |
|  |  |  | Reverse | TCCAGCACAGGACTGCACC |  |
|  |  |  | Sequencing | TCTTTCTCCAGTTGAGC |  |
|  |  |  | Analyzing seq. (Reverse T>C) | C*T/C*GAAGCTCTTCCATCCCCGAGGGCAG |  |
| *ALOX15* | rs34210653 | G>A | Forward | GTCTCCAGCGTTGCATCC | 93 |
|  |  |  | Reverse | (B)-CCTTCCTTAGCTGGACTGGTAC |  |
|  |  |  | Sequencing | GGGGGCAGCCGCAT |  |
|  |  |  | Analyzing seq. (Forward G>A) | C*G/A*TGCAGGGTGCATTAGGCACCCAAG |  |
| *PGIS* | rs5602 | A>G | Forward | ATAATCGTCTCCTGAGCTGCACTT | 145 |
|  |  |  | Reverse | (B)-TTCAAAGAAGGGCCAATTCC |  |
|  |  |  | Sequencing | AGCTGCCTGGTGGGA |  |
|  |  |  | Analyzing seq. (Forward A>G) | GCAC*A/G*TCTTTTCCTTGAGTGTAATTC |  |
| *PGIR* | rs4987262 | G>A | Forward | TCATGAGGGCCAGCAGGAT | 127 |
|  |  |  | Reverse | (B)-AACGGCTCGGTCACCCTC |  |
|  |  |  | Sequencing | TCTGCTGGCGGTACA |  |
|  |  |  | Analyzing seq. (Forward G>A) | TGC*G/A*GCAGAGGCTGAGGGTGACC |  |
| *TXAS1* | rs41708 | A>C | Forward | ATTCTCTCCCTGTCTCCTTGAAAG | 91 |
|  |  |  | Reverse | (B)-TGCCATCAATGGACTAATAGCAAT |  |
|  |  |  | Sequencing | AGGGTTTGTGAGCAATA |  |
|  |  |  | Analyzing seq. (Forward A>C) | A*A/C*CCTTACACCAACATATATTGCTAT |  |
| *TXA2R* | rs1131882 | G>A | Forward | GGCCACGCGCAAGTAGAT | 118 |
|  |  |  | Reverse | (B)-CTCCCCTTTGCAGGTCTTCA |  |
|  |  |  | Sequencing | CAGCACTGTCTGGGC |  |
|  |  |  | Analyzing seq. (Forward G>A) | *G/A*ATGAAGACCTGCAAAGGGGAGAGCT |  |

Forward, forward primer for polymerase chain reaction; Reverse, reverse primer for polymerase chain reaction; (B), biotinylation; Sequencing, sequencing primer for pyrosequencing; Analyzing seq.; target sequence to analyze genotype.

*ALOX5*, 5-lypoxygenase; *ALOX12*, 12-lypoxygenase; *ALOX15*, 15-lypoxygenase; *COX1*, cyclooxygenase 1; *COX2*, cyclooxygenase 2; *CYP2C19*, cytochrome P450 2C19; *PGE2S*, prostaglandin E2 synthase; *PGE2R*, prostaglandin E2 receptor; *PGIS*, prostaglandin I synthase; *PGIR*, prostaglandin I receptor; *TXAS1*, thromboxane A synthase 1; *TXA2R*, thromboxane A2 receptor.

**Supplementary table 2. Analyzing sequences to analyze the genotypes of each gene amplified in multiplex pyrosequencing**

| Gene order of sequencing | Multiplex type | Annealing temperature | Analyzing sequence* |
| --- | --- | --- | --- |
| *CYP2C19**2-*CYP2C19**3 | Duplex | 56 | TCC*C/T*AT*C/T*CGGGAAAATA |
| *COX2-COX1-ALOX5* | Triplex | 56 | *G/C*CCAA*G/A*GCC*T/C*TCACCATC |
| *ALOX12-TXA2R-PGIR* | Triplex | 58 | TC*T/C*GG*G/A*AAAGCC*G/A*GCTTGC |
| *TXAS1-ALOX15-PGIS* | Triplex | 56 | A*A/C*CCCG*G/A*CAC*A/G*TTTTCACAGTTTT |

*Italic letters indicate genotypes to be analyzed for each gene amplified in the duplex or triplex PCR product.

*ALOX5*, 5-lypoxygenase; *ALOX12*, 12-lypoxygenase; *ALOX15*, 15-lypoxygenase; *COX1*, cyclooxygenase 1; *COX2*, cyclooxygenase 2; *CYP2C19*, cytochrome P450 2C19; *PGIS*, prostaglandin I synthase; *PGIR*, prostaglandin I receptor; *TXAS1*, thromboxane A synthase 1; *TXA2R*, thromboxane A2 receptor.

**Supplementary table 3. Primer sequences for the bisulfite pyrosequencing for target genes**

| Genes | Primers | Primer sequence (5'->3') | Annealing temperature | PCR product (bp) |
| --- | --- | --- | --- | --- |
| *COX1* | PCR primer (forward) | GGGGTTGGGTAGAGGAAGT | 54 | 236 |
|  | PCR primer (reverse) | (B)-ACATTCAAAACTCCACCAAAAAAC |  |  |
|  | Sequencing primer | GGGAGGGGATGGGTT |  |  |
|  | Analyzing seq | GGAGTTTYGGGTAGTGTGYGAGGYGTAYGTATAGGAGTTTGTATTTTG |  |  |
| *COX2* | PCR primer (forward) | AGATTTTTGGAGAGGAAGTTAAGT | 58 | 199 |
|  | PCR primer (reverse) | (B)-AAATAATCCCCACTCTCCTATC |  |  |
|  | Sequencing primer | AAAGATTGAGAAGAAGAAAAGATAT |  |  |
|  | Analyzing seq | TTGGYGGAAATTTGTGYGTTTGGGGYGGTGGAATTYGGGGAGGAGAGG |  |  |
| *ALOX5* | PCR primer (forward) | GAATGGATGAGGGGTGGTA | 62 | 154 |
|  | PCR primer (reverse) | (B)-CAATACTTCTCTCCCACTCTTC |  |  |
|  | Sequencing primer | ATTTAGGAGAGAGGTTTTTG |  |  |
|  | Analyzing seq | TTTYGTTYGAGGYGAGGTTTYGTTTAGTYGGYGTYGYGTGAAGAGTGGG |  |  |
| *ALOX12* | PCR primer (forward) | GTTGTGGGGATTGGTTGT | 58 | 225 |
|  | PCR primer (reverse) | (B)-CTCATCCCCTACTTCCTCC |  |  |
|  | Sequencing primer | GAGTTAGGGTGAGGTTAA |  |  |
|  | Analyzing seq | YGGGGTGGGTGYGGYGGGATTTTTTTGGATTTTTYGGGAAGGYGGGATYGA |  |  |
| *ALOX15* | PCR primer (forward) | (B)-TAGGAGGAGTTGATTGGAGATTT | 60 | 143 |
|  | PCR primer (reverse) | AAAAACCCATCTTACTCAAAAATATT |  |  |
|  | Sequencing primer | TAAATAACCCAACCTAAAAC |  |  |
|  | Analyzing seq | CCCRCCCTCTCRAAAATCRCTTAAAATCRAAATATAAATAACAAATCTCCAATC |  |  |
| *PGES2* | PCR primer (forward) | GGGTGAGGGAGAAGTTAAGGGAATTTTT | 56 | 157 |
|  | PCR primer (reverse) | (B)-AATCACTACCCAAAAACCTTTAAAA |  |  |
|  | Sequencing primer | GTGTGTTAGGAGTTTAAGTT |  |  |
|  | Analyzing seq | YGAAAYGTTYGTTAGAGTYGTAGAGGTTYGTTYGGGAAYGTTTGTAGATYG |  |  |
| *PGER2* | PCR primer (forward) | GGAGGGAGAGAAGGAGTTTG | 56 | 192 |
|  | PCR primer (reverse) | (B)-TCCCTCCCAACCCTCTACTA |  |  |
|  | Sequencing primer | TGTAGGTGTATTGAAGTTGT |  |  |
|  | Analyzing seq | TYGYGATYGGYGGGAGYGGYGTTTTTYGTTTTTTYGGGGTTYGGTATTYGAAG |  |  |
| *PGIS* | PCR primer (forward) | (B)-TTTTATTTGGGAGTGGGTTAGG | 56 | 210 |
|  | PCR primer (reverse) | AAACCCTTAAACTACAACCC |  |  |
|  | Sequencing primer | CAACAACAACAACAATAC |  |  |
|  | Analyzing seq | RACCAAAAAA CCRAAAAACR CRACCCAAAC CATCRCRAAA CT |  |  |
| *PGIR* | PCR primer (forward) | (B)-GGAATTTTTTAGTTTAGATTTGGGATG | 56 | 228 |
|  | PCR primer (reverse) | ACTCAAAAAACTAATACCCAACAAATC |  |  |
|  | Sequencing primer | CCAATCCCATCACCAACAC |  |  |
|  | Analyzing seq | CRCRAAAACC RAAAAACRCR CCRATCRCCR TACRCTCAAA A |  |  |
| *TXAS1* | PCR primer (forward) | GGGGTAGGGAGAGTTATAGGGA | 56 | 191 |
|  | PCR primer (reverse) | (B)-AAACCCACCCTCTCACCCACT |  |  |
|  | Sequencing primer | GGAGAGTTATAGGGAGTT |  |  |
|  | Analyzing seq | YGYGYGTAATYGAGYGTYGATGGTAYGGATGYGAGGYGGGG |  |  |
| *TXA2R* | PCR primer (forward) | TGGAAGGGGTTTTAAATATGTTTTGT | 56 | 227 |
|  | PCR primer (reverse) | (B)-CCAACCCCCTACCAAACCTCTT |  |  |
|  | Sequencing primer | GTTGTTTAGATGGTGAATAAT |  |  |
|  | Analyzing seq | GYGGGTTTGGYGGYGGGAGYGTYGGGAAGA |  |  |

Forward, forward primer for polymerase chain reaction; Reverse, reverse primer for polymerase chain reaction; (B), biotinylation; Sequencing, sequencing primer for pyrosequencing; Analyzing seq.; target sequence to analyze methylation status.

*ALOX5*, 5-lypoxygenase; *ALOX12*, 12-lypoxygenase; *ALOX15*, 15-lypoxygenase; *COX1*, cyclooxygenase 1; *COX2*, cyclooxygenase 2; *CYP2C19*, cytochrome P450 2C19; *PGE2S*, prostaglandin E2 synthase; *PGE2R*, prostaglandin E2 receptor; *PGIS*, prostaglandin I synthase; *PGIR*, prostaglandin I receptor; *TXAS1*, thromboxane A synthase 1; *TXA2R*, thromboxane A2 receptor.

**Supplementary table 4. Allele frequency and Hardy–Weinberg equilibrium analysis of the target genes**

| Gene name | Chromosome | Reference SNP number | Allele type | | Minor allele frequency |  | Hardy-Weinberg equilibrium analysis | | | |
| --- | --- | --- | --- | --- | --- | --- | --- | --- | --- | --- |
|  |  |  | Reference | Alternative |  | Genotypes | Patient. number | Observed heterozygosity | Expected heterozygosity | p-value |
| *ALOX5* | 10 | rs745986 | A | G | 0.196 | GG/GA/AA | 651/309/42 | 0.308 | 0.315 | 0.483 |
| *ALOX12* | 17 | rs1126667 | G | A | 0.279 | GG/GA/AA | 721/2/279 | 0.002 | 0.403 | <0.001 |
| *ALOX15* | 17 | rs34210653 | G | A | <0.001 | GG/GA/AA | 1002/0/0 | <0.001 | <0.001 | 1.000 |
| *COX1* | 9 | rs3842788 | G | A | 0.066 | GG/GA/AA | 873/126/3 | 0.126 | 0.123 | 0.797 |
| *COX2* | 1 | rs20417 | C | G | 0.057 | CC/CG/GG | 891/108/3 | 0.108 | 0.107 | 1.000 |
| *CYP2C19*2* | 10 | rs4244285 | G | A | 0.292 | GG/GA/AA | 507/404/91 | 0.403 | 0.414 | 0.445 |
| *CYP2C19*3* | 10 | rs4986893 | G | A | 0.098 | GG/GA/AA | 811/185/6 | 0.185 | 0.177 | 0.282 |
| *PGIS* | 20 | rs5602 | A | G | 0.362 | AA/AG/GG | 420/438/144 | 0.437 | 0.462 | 0.088 |
| *PGIR* | 19 | rs4987262 | G | A | 0.029 | GG/GA/AA | 948/50/4/ | 0.050 | 0.056 | 0.008 |
| *TXAS1* | 7 | rs41708 | C | A | 0.289 | CC/AC/AA | 507/410/85 | 0.409 | 0.411 | 0.878 |
| *TXA2R* | 19 | rs1131882 | G | A | 0.387 | GG/GA/AA | 383/462/157 | 0.461 | 0.477 | 0.389 |
|  |  |  |  |  |  |  |  |  |  |  |

SNP, single nucleotide polymorphism.

*ALOX5*, 5-lypoxygenase; *ALOX12*, 12-lypoxygenase; *ALOX15*, 15-lypoxygenase; *COX1*, cyclooxygenase 1; *COX2*, cyclooxygenase 2; *PGE2S*, prostaglandin E2 synthase; *PGE2R*, prostaglandin E2 receptor; *PGIS*, prostaglandin I synthase; *PGIR*, prostaglandin I receptor; *TXAS1*, thromboxane A synthase 1; *TXA2R*, thromboxane A2 receptor.

**Supplementary table 5. Variables and number of missing values to be imputed**

| Variable groups | Variables | Patient number |
| --- | --- | --- |
| Blood tests | Homocysteine | 213 (21.6%) |
|  | hsCRP | 215 (21.8%) |
|  | Fibrinogen | 169 (17.1%) |
|  | Total cholesterol | 11 (1.1%) |
|  | LDL | 18 (1.8%) |
|  | HDL | 23 (2.3%) |
|  | HbA1c | 23 (2.3%) |
|  | Creatinine | 8 (0.8%) |
|  | Triglyceride | 48 (4.9%) |
| Platelet function test | PRU | 3 (0.3%) |
| Mediators | TXB2 | 18 (1.8%) |
|  | PGE2 | 2 (0.2%) |
|  | 6-keto-PGF1α | 17 (1.7%) |
|  | LTB4 | 18 (1.8%) |
|  | LXA4 | 18 (1.8%) |
| Promoter methylations | *PGIR* | 1 (0.1%) |
| Total |  | 806 |

6-ketoPGF1α, 6-keto prostaglandin F1α; HbA1c, hemoglobin A1c; HDL, high density lipoprotein; hsCRP, high sensitivity C-reactive protein; LDL, low density lipoprotein cholesterol; LTB4, leukotriene B4; LXA4, lipoxin A4; PGE2, prostaglandin E2; *PGIR*, prostaglandin I receptor; PRU, P2Y12 reaction unit; TXB2, thromboxane B2.

**Supplementary table 6. Comparisons of aspirin reaction unit (ARU) and P2Y12 reaction unit (PRU) levels with other variables**

| Variable groups | Variables | | ARU | PRU |
| --- | --- | --- | --- | --- |
| Cardiovascular risk factors | Age^a^ | | .069^*^ | .186^***^ |
|  | Sex (men [n = 644]:women [n = 344])^b^ | | 476.2 ± 55.6:474.9 ± 60.0 | 182.4 ± 52.0:205.3 ± 54.9^***^ |
|  | Hypertension (yes [n = 589]no [n = 399])^b^ | | 476.9 ± 56.9:473.9 ± 57.5 | 192.0 ± 54.5:188.0 ± 53.5 |
|  | Diabetes (yes [n = 312]:no [n = 676])^b^ | | 477.5 ± 58.9:474.9 ± 56.3 | 192.5 ± 54.0:189.4 ± 54.2 |
|  | Smoking (yes [n = 259]:no [n = 729])^b^ | | 472.6 ± 56.6:476.8 ± 57.3 | 177.6 ± 51.6:194.9 ± 54.3^***^ |
| Blood tests^a^ | GPT (U/L) | | -0.010 | -.0650^*^ |
|  | GOT (U/L) | | -0.025 | -0.047 |
|  | Blood urea nitrogen (mg/dL) | | 0.007 | .0660^*^ |
|  | Creatinine (mg/dL) | | 0.024 | 0.036 |
|  | Homocysteine | | 0.026 | -0.031 |
|  | Fibrinogen (mg/dL) | | -0.033 | -0.018 |
|  | Total cholesterol (mg/dL) | | -.080^*^ | -.0770^*^ |
|  | LDL (mg/dL) | | -.0710^*^ | -.0810^*^ |
|  | HDL (mg/dL) | | -0.020 | -0.034 |
|  | Triglyceride (mg/dL) | | -0.031 | 0.021 |
|  | hsCRP (mg/L) | | 0.000 | 0.011 |
|  | White blood cells (/μL) | | -0.087** | -0.037 |
|  | Hemoglobin (g/dL) | | -0.020 | -0.437*** |
|  | Platelet(10^3^/μL) | | -0.114*** | -0.073* |
|  | Hemoglobin A1c (%) | | -0.017 | -0.026 |
| Cyclooxygenase and lipoxygenase mediators^a^ | LTB4 | | -0.123*** | 0.039 |
|  | LXA4 | | 0.002 | 0.046 |
|  | TXB2 | | 0.034 | -0.009 |
|  | PGE2 | | -0.005 | 0.042 |
|  | 6-keto-PGF1α | | 0.027 | 0.060 |
| Single nucleotide polymorphisms (mean±SD)^b^ | *CYP2C19**2 (rs4244285) | G/G (n = 500) | 474.4 ± 56.0 | 176.3 ± 56.4*** |
|  |  | G/A (n = 399) | 476.8 ± 57.4 | 201.1 ± 47.9*** |
|  |  | A/A (n = 89) | 477.9 ± 61.9 | 220.8 ± 42.3*** |
|  | *CYP2C19**3 (rs4986893) | G/G (n = 800) | 476.0 ± 58.4 | 186.6 ± 54.1*** |
|  |  | G/A (n = 182) | 473.6 ± 51.1 | 206.0 ± 51.8*** |
|  |  | A/A (n = 6) | 491.0 ± 55.1 | 212.0 ± 30.4*** |
|  | *COX1* (rs3842788) | G/G (n = 860) | 475.5 ± 58.0 | 190.5 ± 54.6 |
|  |  | G/A (n = 125) | 477.7 ± 50.4 | 189.3 ± 50.7 |
|  |  | A/A (n = 3) | 447.6 ± 57.7 | 179.1 ± ±65.0 |
|  | *COX2* (rs20417) | C/C (n = 880) | 476.8 ± 56.1 | 191.0 ± 53.7 |
|  |  | C/G (n = 105) | 466.3 ± 64.5 | 184.1 ± 56.9 |
|  |  | G/G (n = 3) | 479.8 ± 62.4 | 222.5 ± 31.1 |
|  | *ALOX5* (rs745986) | A/A (n = 642) | 473.0 ± 57.8 | 191.2 ± 52.9 |
|  |  | A/G (n = 305) | 479.4 ± 54.1 | 190.0 ± 55.4 |
|  |  | G/G (n = 41) | 490.6 ± 65.3 | 180.3 ± 61.6 |
|  | *PGIS* (rs5602) | A/A (n = 415) | 475.2 ± 59.0 | 189.2 ± 53.7 |
|  |  | A/G (n = 431) | 476.6 ± 55.3 | 191.1 ± 54.3 |
|  |  | G/G (n = 142) | 474.6 ± 57.4 | 191.4 ± 54.6 |
|  | *PGIR* (rs4987262) | G/G (n = 934) | 476.0 ± 57.1 | 189.3 ± 53.9* |
|  |  | G/A (n = 50) | 470.5 ± 54.5 | 210.0 ± 52.5* |
|  |  | A/A (n = 4) | 482.7 ± 91.7 | 189.6 ± 74.4* |
|  | *TXAS1* (rs41708) | C/C (n = 500) | 476.6±58.4 | 189.2±54.8 |
|  |  | A/C (n = 403) | 475.8±56.6 | 190.8±53.3 |
|  |  | A/A (n = 85) | 470.5±52.3 | 194.9±54.1 |
|  | *TXA2R* (rs1131882) | G/G (n = 154) | 483.6 ± 60.2 | 192.5 ± 55.8 |
|  |  | G/A (n = 458) | 476.3 ± 57.4 | 189.6 ± 53.7 |
|  |  | A/A (n = 376) | 471.8 ± 55.1 | 190.4 ± 54.0 |
| Promoter methylation^a^ | *COX1* | | 0.131*** | -0.001 |
|  | *COX2* | | 0.013 | -0.015 |
|  | *ALOX5* | | 0.052 | 0.047 |
|  | *ALOX12* | | 0.025 | 0.001 |
|  | *ALOX15* | | -0.061 | -0.001 |
|  | *PGE2S* | | 0.095** | 0.032 |
|  | *PGE2R* | | 0.076* | -0.032 |
|  | *PGIS* | | 0.038 | -0.014 |
|  | *PGIR* | | 0.024 | 0.012 |
|  | *TXAS1* | | 0.065^*^ | 0.058 |
|  | *TXA2R* | | 0.019 | -0.031 |

^a^Pearson correlation coefficient between the ARU or PRU and each variable

^b^Average (± SD) comparison between the groups classified for each variable

*p < 0.05; **p < 0.01; ***p < 0.001.

6-ketoPGF1α, 6-keto prostaglandin F1α; *ALOX5*, 5-lypoxygenase; *ALOX12*, 12-lypoxygenase; *ALOX15*, 15-lypoxygenase; *COX1*, cyclooxygenase 1; *COX2*, cyclooxygenase 2; *CYP2C19*, cytochrome P450 2C19; GOT, glutamic oxaloacetic transaminase; GPT, glutamic pyruvate transaminase; HDL, high-density lipoprotein; hsCRP, high sensitivity C-reactive protein; LDL, low-density lipoprotein; LTB4, leukotriene B4; LXA4, lipoxin A4; PGE2, prostaglandin E2; *PGE2S*, prostaglandin E2 synthase; *PGE2R*, prostaglandin E2 receptor; *PGIS*, prostaglandin I synthase; *PGIR*, prostaglandin I receptor; *TXAS1*, thromboxane A synthase 1; *TXA2R*, thromboxane A2 receptor; TXB2, thromboxane B2.

**Supplementary figure 1.** **Promoter CpG islands and regions for pyrosequencing of the 11 target genes.** Open *bar*: exon regions; closed *bar*: the regions targeted for bisulfite pyrosequencing; open *bar in the middle of the closed bar*: sequencing primer region; *arrow*: transcription start site of each gene. *ALOX5*, 5-lipoxygenase; *ALOX12*, 12-lipoxygenase; *ALOX15*, 15-lipoxygenase; *COX1*, cyclooxygenase 1; *COX2*, cyclooxygenase 2; *PGE2S*, prostaglandin E2 synthase; *PGE2R*, prostaglandin E2 receptor; *PGIS*, prostaglandin I synthase; *PGIR*, prostaglandin I receptor; *TXAS1*, thromboxane A synthase 1; *TXA2R*, thromboxane A2 receptor.


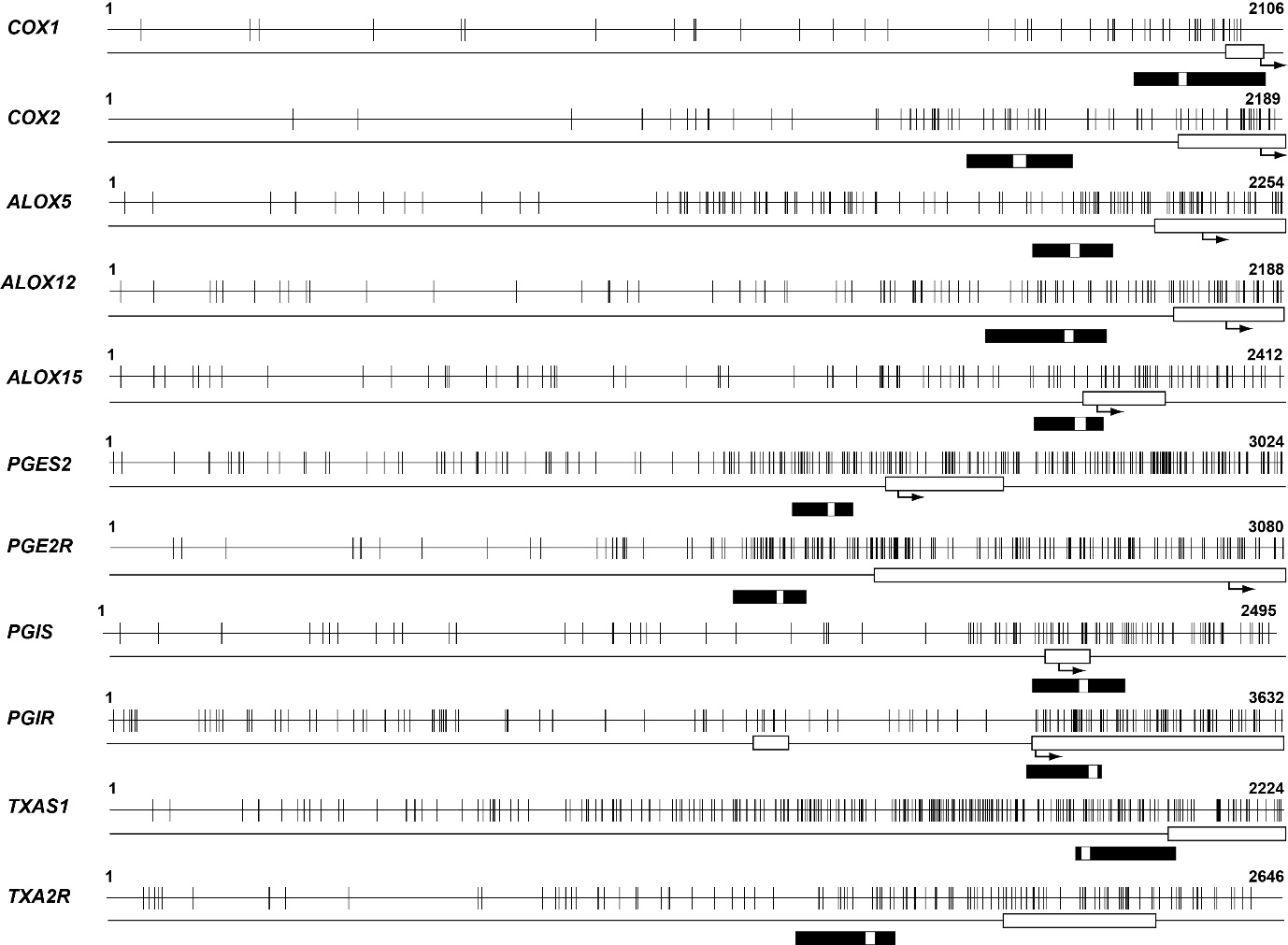

Supplement: Supplementary file 1 — Supplementary Material 1. [file 12920_2024_1936_MOESM1_ESM.docx]
